# Block copolymers: controlling nanostructure to generate functional materials – synthesis, characterization, and engineering

**DOI:** 10.1039/c5sc03505h

**Published:** 2016-01-13

**Authors:** Thomas H. Epps, III, Rachel K. O'Reilly

**Affiliations:** a Department of Chemical and Biomolecular Engineering and Department of Materials Science and Engineering , University of Delaware , Newark , Delaware 19716 , USA . Email: thepps@udel.edu; b Department of Chemistry , University of Warwick , Gibbet Hill , Coventry , CV4 7AL , UK . Email: rachel.oreilly@warwick.ac.uk

## Abstract

In this perspective, we survey recent advances in the synthesis and characterization of block copolymers, discuss several key materials opportunities enabled by block copolymers, and highlight some of the challenges that currently limit further realization of block copolymers in promising nanoscale applications. One significant challenge, especially as the complexity and functionality of designer macromolecules increases, is the requirement of multiple complementary techniques to fully characterize the resultant polymers and nanoscale materials. Thus, we highlight select characterization and theoretical methods and discuss how future advances can improve understanding of block copolymer systems. In particular, we consider the application of theoretical/simulation methods to the rationalization, and prediction, of observed experimental self-assembly phenomena. Finally, we explore several next steps for the field and emphasize some general areas of emerging research that could unlock additional opportunities for nanostructure-forming block copolymers in functional materials.

## Abstract 2

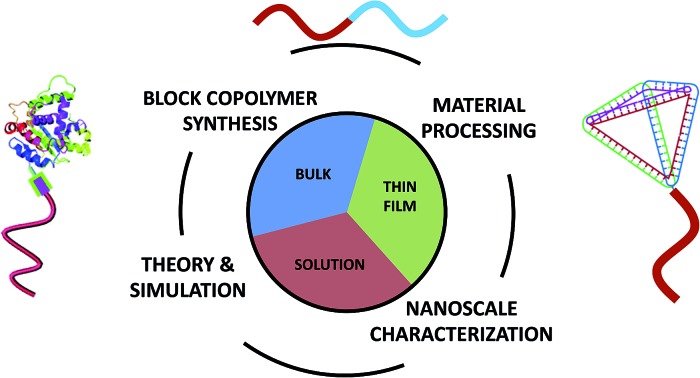
In this perspective, we survey recent advances in the synthesis and characterization of block copolymers, discuss several key materials opportunities enabled by block copolymers, and highlight some of the challenges that currently limit further realization of block copolymers in promising nanoscale applications.

## Introduction to block copolymers

Nature uses molecular self-assembly to create precision nanostructures, craft unique compartmentalized environments, and build large constructs through hierarchical assembly. Indeed, recent developments in nanotechnology have mimicked natural approaches by utilizing nucleic acid sequence specificity to create higher order structures through the creation of DNA origami tiles, other three-dimensional structures, and nanomechanical molecular devices.^1^ Inspired by these recent advances and motifs, considerable efforts have been initiated to recreate such concepts using synthetic building blocks such as small-molecule surfactants and block copolymers (BCPs).

**Fig. 1:**
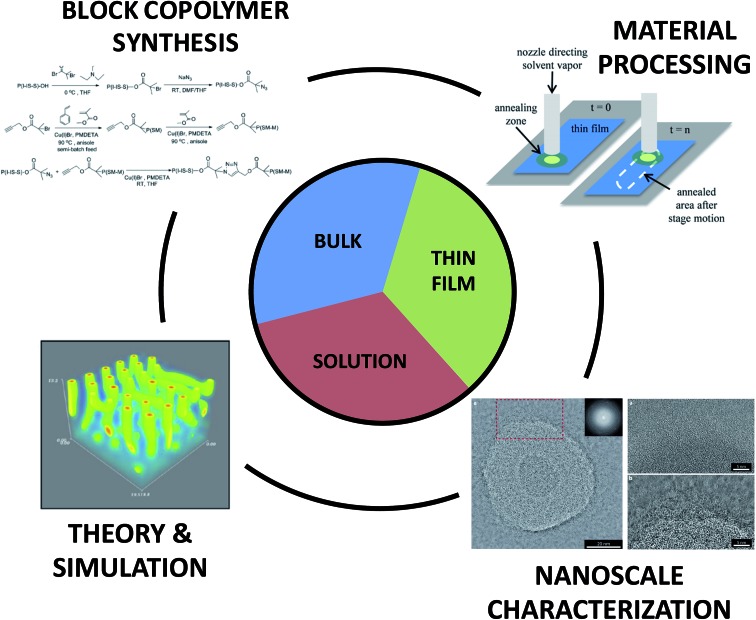
Key aspects of block copolymer materials design. The synthesis, material processing, nanoscale characterization, and theory/simulation of block copolymers are all crucial in the development of new hierarchically assembled structures. These factors are intimately linked to the informed design of materials for bulk, thin film, and solution applications. Top right image – reprinted with permission from ref. 89 © American Chemical Society; bottom right image – reprinted with permission from ref. 47 © Royal Society of Chemistry; bottom left image – reprinted with permission from ref. 88 © American Chemical Society; top left image – reprinted with permission from ref. 77 © American Chemical Society.

Small-molecule surfactants and BCPs are both fashioned from two or more chemically dissimilar constructs that are covalently-bonded into a single molecule. Through a delicate mix of molecular interactions and materials processing, these molecules form a variety of nanoscale structures. The linking of constitutionally different units permits the combination of distinct properties within a macromolecule and enables interesting nanoscale assembly phenomena, and ultimately, unique macroscale behavior.^2^ Importantly, many of the unique physical properties inherent to BCP materials are a result of the nanoscale hybridization of their components and cannot be accessed through simple blending of non-bonded blocks. Furthermore, the increased number of repeat units in BCPs, compared to small molecules, leads to dramatically improved morphological stability. This stability provides significant opportunities for BCP utilization in a broad range of environments but also necessitates new methodologies to control the precise assembly of organized nanostructures. Indeed, through advances in polymer synthesis, functionalization, processing, and characterization, it is now possible to design, fabricate, and explore a vast array of BCPs with diverse and sophisticated self-assembly potential in bulk, thin film, and dilute solution environments. In Fig. 1 for example, the combination of multiple polymerization methods and targeted coupling chemistries enables the synthesis of complex macromolecules such as tapered BCPs (top left), the use of solvent processing recipes on motorized stages permits the generation of directed nanostructures for thin film templating (top right), the cryogenic transmission electron microscopy of cylindrical BCP micelles allows one to visualize nanostructure formation in solution environments (bottom right), and self-consistent field theory simulations of solvent removal in a cylinder-forming BCP thin film informs structure/processing relationships (bottom left). *(We also note that many hydrogel and concentrated solution systems contain BCPs, but discussion of those materials is not included in this work.)*

### Bulk

Bulk assembly has been studied extensively for over 50 years, and the phase behavior of traditional A–B diblock copolymers is well-researched both theoretically and experimentally. In conventional bulk materials self-assembly processes are governed by an unfavorable mixing enthalpy coupled with entropic losses due to macromolecular junctions and chain stretching. Current commercial applications of bulk BCPs (diblock and multiblock) include thermoplastic elastomers for gaskets, cable insulation, footwear, blending, adhesives, automotive bumpers, snowmobile treads, *etc.* (*e.g.* Kraton™, Styroflex™, Solprene™, Hytrel™, Engage™, Sofprene™); thermoplastics for medical devices, protective headgear, and piping systems; and elastomers for car tires (*e.g.* poly(styrene-*b*-isoprene-*b*-butadiene) rubber [SIBR] from Goodyear) among others. For the case of thermoplastic elastomers, BCPs enable the facile and low-cost generation of a myriad of application-specific recyclable, flexible, thermoformed (or blow-moldable), creep-resistant, and durable materials as compared to conventional thermosets, primarily as a result of prescribed nanoscale phase separation.

Well-defined BCPs can phase separate into a variety of periodic and nanoscale morphologies according to the relative composition of the blocks, the overall degree of polymerization (*N*), the polymer–polymer interaction parameter (*χ*), and the ratio of statistical segment lengths.^3^ The conceptual ability to tune morphology through adjustments in chemical composition allows one to generate materials tailored toward thermoplastic elastomer, membrane, and other applications. Though the mechanism underlying nanostructure formation for the simplest classes of BCPs with non-specific interactions is well understood, new macromolecular designs that incorporate multiblocks,^4^ copolymer mixtures,^5^ specific interactions (hydrogen-bonding,^6^ π–π stacking,^7^ *etc.*), engineered dispersity,^8^ tapered segment profiles,^9^ sequence-controlled monomer distributions,^10^ small-molecule dopants (*e.g.* salts, plasticizers, and inorganic precursors),^11–13^ and functional end-groups^14^ significantly complicate the understanding of bulk macromolecular assembly, yet potentially yield exciting opportunities for novel material designs such as active membranes for batteries and fuel cells,^15^ catalyst supports/scaffolds,^16,17^ actuators, and self-healing or shape-memory systems.^18,19^

### Thin film

In addition to the factors that affect assembly in bulk, self-assembly processes in thin films (<several hundred nanometers in thickness) are strongly influenced by surface energetics and film thickness (commensurability) considerations.^20^ The incorporation of these additional driving forces, as well as significant processing history effects due to kinetic limitations associated with thin film assembly, has a substantial impact on nanostructure formation and surface topology in thin films. Significant recent progress has been made in manipulating BCP thin film morphologies for media storage, photonics, nanolithography, nanotemplating, and ultrafiltration applications. A few examples for which thin film BCPs have received interest in high value industrial processing are: conventional chip manufacturing,^21^ nanotemplating for dense bit-patterned media that could facilitate doubling of hard disk drive densities,^22^ and nanolithography for patterning next-generation semiconductor devices.^23^ Despite these industrial endeavors, each with its own challenges,^24^ distinct hurdles that limit wide-spread usage of BCP thin films in emerging technologies remain.^25^ These hurdles include precise control over the directed assembly of nanoscale domains through cost-effective and scalable approaches, understanding the influence of nanostructure formation dynamics and processing protocols (such as the influence of various annealing recipes, film casting methods, surface energetics, and polymer molecular weights and architectures) on morphology and orientation, elimination (or significant reduction) of defects, and translation of nanopatterning techniques to non-traditional substrates (*e.g.* flexible substrates, porous scaffolds, graphene, metals).^24,26,27^ A variety of surface fields, thermal and solvent annealing (uniform, gradient, and zone-annealing) protocols,^26^ and other external fields (*e.g.* magnetic, electric, mechanical, *etc.*)^28–30^ have been employed to manipulate nanoscale morphology, orientation, and ordering in BCP thin films. Further understanding of the many parameters associated with these tools will permit additional BCP applications in coatings,^31^ nanoporous membranes,^32^ anti-fouling materials,^33^ and analytical and process-scale separation membranes,^34^ electronics,^35,36^ and optoelectronics,^7,37^ including complex circuits,^38,39^ stretchable/flexible electronics,^40^ optic and acoustic wave guides,^41^ sensors,^42^ and holographic arrays:^43^ all areas in which three-dimensional or hierarchical structures can transform materials design.^32^

Surface grafted (brush) block copolymers also are receiving significant interest as nanostructured surface coatings for drag reduction, surface energy modification, biosensing, and cellular manipulation applications.^44,45^ These brush systems incorporate an additional variable in terms of block order (even in diblock copolymer systems) as a consequence of attachment to a substrate and typically do not possess the same kinetic limitations inherent in BCP films. Some key challenges include high-throughput synthesis and characterization of block copolymer brush systems with high reproducibility and accuracy, generating a detailed knowledge-base of factors that influence phase behavior and surface properties, and adapting block copolymer brush systems for specific applications.^44,45^

### Dilute solution

Dilute solution self-assembly is similarly complex because the introduction of a single solvent, or multiple solvents, dramatically affects the assembly process and the resultant nanostructures.^2,46^ This added complexity is largely due to additional surface tension, interaction parameter, and entropy effects that can significantly alter the formation and stability of macromolecular assemblies in solution.^44,47^ Thermodynamic and kinetic constraints can lead to the formation of various aggregates such as micelles and vesicles, related to the spherical, cylindrical, and lamellar morphologies, which are found in bulk systems;^2,48^ although exotic structures such as helices,^49^ toroids,^50^ and networks^48,50^ also have been reported. The majority of research has focused on aqueous self-assembly, for which the driving force is primarily hydrophobic interactions; however, there have been significant efforts to examine BCP assembly in organic solvents,^51^ ionic liquids,^52^ supercritical solvents,^53^ mixed solvents, and during polymerization.^54^ The free-energy landscape, as accessed through assembly protocols, solution processing (*e.g.* agitation and shear), hydrogen-bonding, reversible and irreversible stimuli-responses, salts, and cross-linking are critical in determining the final state of solution assemblies.^44,46,55^ Several key advantages of BCP solution assemblies (as opposed to low molecular weight surfactant assemblies) include low critical aggregation concentrations (CAC)s and slow inter-aggregate chain exchange in highly selective solvents such as water.^2^ Additionally, high loading capacities coupled with the ability to incorporate a myriad of functionalities and BCP compositions and architectures enables efficient bottom-up strategies to synthesize surfactants for interfacial stabilization such as commercially-relevant Pluronics™ and Tetronics™ and nanocontainers for biological (therapeutic agent delivery, imaging, diagnostics, theranostics) catalysis, separations, and self-healing applications.^44,46,56^ Unfortunately, the slow dynamics that normally are advantageous in producing stable nanocarriers also lead to kinetically trapped structures,^46,57^ thus such systems require careful optimization of preparation conditions to produce well-defined, uniform, and reproducible solution assemblies.

**Fig. 2:**
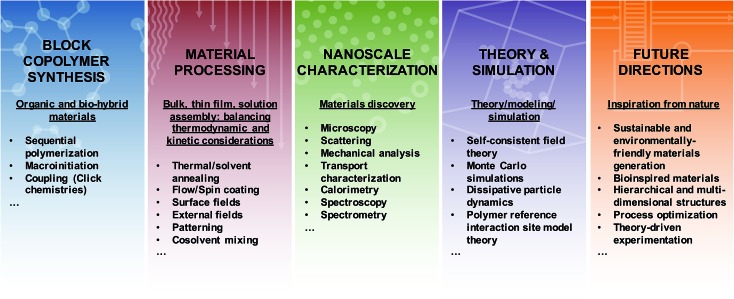
Generating functional materials from block copolymers. Highlighted features of block copolymers are categorized into synthetic approaches to produce novel materials, processing routes to manipulate nanoscale arrangement, characterization methods to obtain detailed nanoscale information, theoretical and simulation-based approaches to improve fundamental understanding, and directions for future research.

Overall, the substantial need for new materials with well-defined and predictable nanoscale and macroscale characteristics has stimulated further study of macromolecular assemblies in bulk, thin film, and solution environments, as all arenas are poised to engender ground-breaking technological and societal impacts. The on-going fabrication of more exotic, hierarchical, and nature-inspired BCPs provides tantalizing glimpses toward emerging applications enabled through complex morphology generation; however, further efforts linking synthesis, nanostructure fabrication, processing, characterization, and theory (see Fig. 2) are necessary to unlock the full potential of BCPs. In the following sections, we describe various methods for the synthesis of BCPs, highlight key tools that enable nanoscale characterization of self-assembling soft materials, discuss select contributions that link experiment with theory, simulation, and modelling, and emphasize several emerging directions for BCP activities. The focus is on recent literature and is not meant to ignore the wealth of seminal investigations that provide the inspiration for the work discussed herein. For the sake of brevity, much of the foundational work can be found in the references sections of the literature highlighted in each of the topics below. Additionally, the examples provided below are not meant to be all-inclusive, but instead are selected to provide a snapshot of the power and challenges associated with experimental and theoretical development of BCP nanostructures for wide-ranging materials.

## Synthesis of block copolymers – designer macromolecules with unique properties

While, the synthesis of BCPs is an established field with many major advances over the last 50–60 years, more recent efforts in controlled polymer synthesis now enable the preparation of a wide range of BCP architectures including, linear, graft, dendritic, star-like, bottle-brush, hyperbranched, and cyclic BCPs.^4^ All of these macromolecular structures have unique and interesting self-assembly behavior; however, given the diversity of available architectures, this review will focus only on the versatility of linear BCPs, which still possess a myriad of opportunities to advance functional materials design. Within the sub-class of linear BCPs, various block types have been prepared such as organic and bio-hybrid BCPs. The first type includes a range of organic (non-biological-based) BCPs that can assemble by minimizing the free energy landscape that primarily is influenced by mixing and chain stretching considerations. We also note that significant work has been carried out in BCP systems that have specific interactions such as hydrogen-bonding, metal-binding, π–π stacking, *etc.*, which leads to self-organizing structures with their own intricate features. Many of these materials have been reviewed extensively in the literature,^7,58^ and while of interest, they will not be discussed in exhaustive detail herein. Instead, we will highlight a range of synthetic tools that can be used to prepare specific organic BCPs with unique properties.

**Fig. 3:**
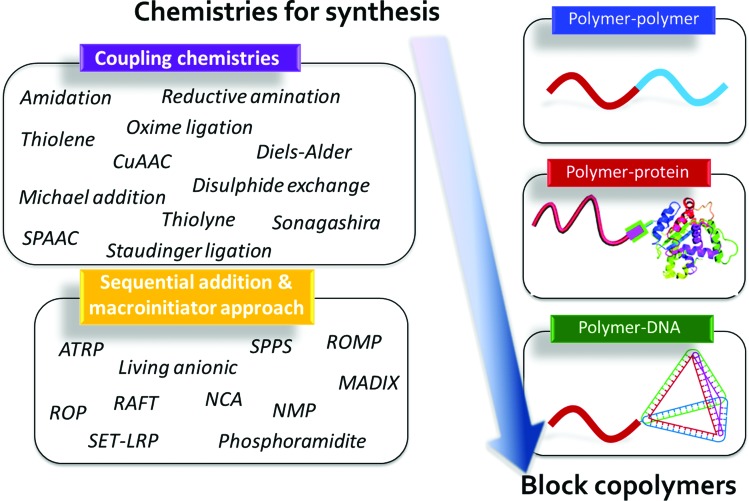
Chemistries for the synthesis of block copolymers. CuAAC, copper-catalyzed azide–alkyne cycloaddition; SPAAC, strain-promoted azide–alkyne cycloaddition; ATRP, atom-transfer radical polymerization; SPPS, solid phase peptide synthesis; ROMP, ring-opening metathesis polymerization; RAFT, reversible addition–fragmentation chain-transfer; ROP, ring-opening polymerization; SET-LRP, single-electron transfer living radical polymerization; NMP, nitroxide-mediated polymerization; NCA, *N*-carboxyanhydride; MADIX, macromolecular design by the interchange of xanthates.

The second type, nature-inspired or bio-hybrid BCPs, forms a burgeoning class of self-assembling materials, in which the potential for secondary structure formation and concerted specific interactions promises unparalleled opportunities in hierarchical and function-driven assembly.^59^ These nature-inspired bio-hybrids often contain at least one constituent derived from a biomolecular building block such as a peptide, protein, nucleic acid, peptoid, or sugars.^60,61^ The combination of synthetic and bio-blocks in a well-defined macromolecule potentially introduces distinct nanostructures, stimuli-responsive character, and specific functions that are difficult to generate in ‘simpler’ organic–organic BCP systems.^59^ As a result, these bio-hybrid BCPs provide opportunities for the realization of diverse and highly targeted applications in self-assembled materials (Fig. 3).

In the following sections, we will highlight several noteworthy advances in the synthesis of linear BCPs that can lead to practical nanoscale assemblies for biomedicine, electronics, catalysis, nanotemplating, and responsive surface materials, among other applications. Furthermore, we also hint at several challenges, such as the need for the sustainable, efficient, and environmentally-friendly generation of functional macromolecules, which will be discussed in greater detail in the emerging directions section of this perspective.

### Methods for synthesis

There are numerous routes toward the synthesis of BCPs, which result in an extraordinarily broad range of macromolecules with tailorable and highly specific properties. These approaches can be broadly divided into three different classes: (continuous) sequential polymerization, macroinitiation, and coupling. Within these methods key considerations include the generation of well-defined polymers at high purity with controlled dispersity and high end-group fidelity, the incorporation of application-specific functional groups, and the ability to combine macromolecules synthesized from diverse polymerization methods, including the coupling of natural and synthetic building blocks. Each of these factors can have marked consequences on macromolecular self-assembly. For example, in many BCP generation approaches the final mixture can contain homopolymer or other “incomplete” contaminants due to loss of end-group fidelity, premature termination, incomplete end-group functionalization, or inefficient coupling. These contaminants can be difficult to remove or quantify; however, they can have a significant influence on macromolecular assembly.^62^ Overcoming these limitations will facilitate copolymer design, as well as provide opportunities for the automated and high-throughput synthesis of complex architectures to possibly enable sustainable materials discovery.^63^

### Continuous sequential polymerization

Perhaps the most straightforward method to prepare BCPs is through continuous sequential polymerization of two or more monomer sets using a single chain growth mechanism. That is to say, controlled polymerization of one monomer, followed by chain extension with a different monomer without intermediate termination/purification steps, can be used to prepare an AB polymer (or through further monomer addition, multiblock systems).^64^ Sequential polymerization is especially applicable to methods such as living ionic, reversible-deactivation radical polymerization, and ring-opening polymerization. In particular, living anionic polymerization is somewhat restricted to this approach due to difficulties in reinitiating a terminated chain end; however, anionic techniques are still desirable for the ability to generate highly uniform polymers of extremely low dispersity and with excellent end-group fidelity,^3^ as demonstrated by Goodyear (SIBR) for tires and by other companies in various applications. Additionally, continuous sequential methodologies can be tuned to produce tapered interfaces between polymer blocks.^9^ These tapered block copolymers represent an emerging class of BCPs with unique and diverse self-assembly behavior.^65^ Reversible-deactivation radical polymerization and ring-opening-based polymers also can be generated through continuous sequential polymerization, but these polymers are amenable to both macroinitiation approaches described below, due to the possibility of reinitiating a dormant chain end. Though sequential polymerization methods are useful for preparing a broad range of BCPs, they are somewhat limited in the polymerization of functional monomers, such as those containing nucleophilic or other reactive functionalities.

One approach to overcome this limitation in functional monomers involves the use of dual initiator (or protected initiator) species such as a hydroxyl-functionalized reversible addition–fragmentation chain-transfer (RAFT) chain transfer agent (CTA), which allows for the orthogonal polymerization of two or more distinct monomers. This route has been used most effectively for the combination of ring-opening and reversible-deactivation radical polymerization methods and unlocks access to a range of functionalizable BCPs.^66^

### Macroinitiator approaches

Although living anionic and anionic ring-opening polymerizations are useful for sequential approaches, the synthetically demanding nature of the reactions typically necessitates a macroinitiator approach to generate BCPs with the desired multiple block functionalities. This macroinitiation route can provide well-defined BCPs, but it normally involves a two-step polymerization process with the possible need for intermediate purification steps.^67^ Fortunately, macroinitiation provides a ready means for generating a highly-defined library of macromolecules for systematic studies. A key consideration is that the polymerization mechanism utilized to synthesize the first block must result in a polymer with excellent end group fidelity to ensure that effective and efficient chain extension is possible.

Two major cases of macroinitiation can be defined; *case one*: the same polymerization mechanism used to generate the macroinitiator is used for chain extension, and *case two*: an orthogonal reaction scheme is used for chain extension. The first case is particularly amenable to ring-opening and reversible-deactivation radical polymerizations, in which the macroinitiator can be re-initiated following intermediate purification. However, challenges still remain in this case. As has been readily demonstrated in the literature, successful BCP formation may still not be possible due to blocking effects, which are based on the reactivity of the macroinitiator towards the chain-extending monomer. Macroinitiator reactivity is especially important when monomers with a more active dormant species (*e.g.* methacrylate or acrylonitrile) are utilized to extend a macroinitiator of lower activity (*e.g.* polystyrene or polyacrylate). This factor can be especially problematic when the specific functionality at the α and ω ends is important and hence reversing the order of polymerization is not possible. Additionally, macroinitiation schemes utilizing a single polymerization mechanism are not always amenable to the preparation of highly amphiphilic and hybrid BCPs, including those containing poly(ethylene oxide), polypeptides, or nucleic acids.

There have been several manipulations to controlled polymerizations to facilitate BCP synthesis from constituent monomers that cannot be polymerized using the same mechanism or initiating scheme (case two). Classic examples are macroinitiators synthesized through anionic or anionic ring-opening polymerization. Polystyrene-*b*-poly(ethylene oxide) [PS-*b*-PEO], polybutadiene-*b*-PEO [PB-*b*-PEO], PEO-*b*-poly(*N*-isopropylacrylamide) [PEO-*b*-PNIPAM], and PEO-*b*-ε-polycaprolactone [PEO-*b*-PCL] are several workhorse synthetic BCPs generated using this route.^48^ Recent efforts have extended this approach to reversible-deactivation radical polymerizations, for which simple and effective chain end modification chemistries enable orthogonal polymerization mechanisms. Additionally, ring-opening metathesis polymerization (ROMP) provides an elegant tool for the synthesis of a diverse range of block copolymers.^68^ ROMP is especially useful for the preparation of BCPs with interesting topologies including cycles,^69^ grafts,^70^ and bottle-brushes.^71^

Two areas that have received recent interest include degradable BCPs, containing polylactide and bio-hybrid systems such as polypeptide BCPs which have possible applications in biomedicine.^72,73^ Additionally, the explosion of click chemistries as a model for polymer functionalization (*e.g.* polymer chain end modification) has facilitated the manipulation of macroinitiators to allow for a second polymerization mechanism. This functionalization avenue has significantly broadened the scope of BCPs accessible through a macroinitiator approach especially for such sustainable and degradable polymers.

**Fig. 4:**
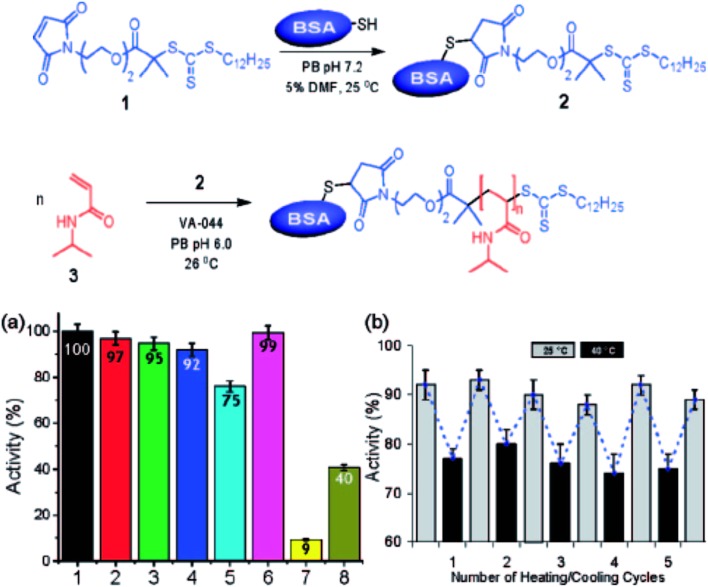
Bio-hybrid block copolymers generated through coupling approaches. (Top image) scheme for the synthesis of protein–polymer BCP. (Bottom image, left) activity of (1) BSA, (2) BSA–macro-CTA, (3) BSA–PNIPAM (free BSA present), (4) BSA–PNIPAM thermal precipitate, (5) BSA–PNIPAM thermal precipitate at 40 °C, (6) BSA physical mixture, (7) PNIPAM, (8) BSA after incubation at 75 °C for 3 h. (Right) activity of BSA–PNIPAM thermal precipitate during thermal cycling between 25 °C and 40 °C [adapted and reprinted with permission from ref. 74 © American Chemical Society].

Macroinitiator approaches also are very effective for the preparation of nature-derived BCPs, especially those from natural biopolymers such as cellulose, chitin, or proteins. Although these methods often lead to more complex architectures instead of simple and low dispersity linear BCPs, the manipulation of natural biopolymers to incorporate selective single site modifications has shown promise.^74^ This method has been demonstrated for the modification of proteins through selective introduction of a functionality (*e.g.* a polymerization initiator) to enable polymerization of a second polymer in a ‘grafting from’ approach and to afford a bio-hybrid BCP (Fig. 4).^74^ To extend this approach and ensure effective and high-yielding BCP synthesis, further efforts are required to selectively incorporate functionalities that enable BCP generation in nature-inspired or nature-derived systems.

### Post-polymerization coupling

Perhaps the most versatile method for BCP synthesis is the post-polymerization coupling approach, which enables the conjugation of blocks with very distinct chemistries. This avenue for linking pre-synthesized macromolecules has been facilitated by the exploitation of click chemistries. The concept was introduced in 2001 by Sharpless,^75^ and was later applied in BCP synthesis,^76^ Click is exemplified by the copper(i)-catalyzed azide–alkyne cycloaddition reaction (CuAAC). This reaction, and other high-yielding and highly-efficient coupling approaches,^14^ allow for the conjugation of a diverse array of end-functionalized polymers to afford a range of BCPs. One challenge in the translation of small molecule click approaches to polymer–polymer conjugation is the requirement for complete end group retention (and/or subsequent functionalization), which has been difficult to achieve for the vast majority of polymerization routes. However, methods such as living anionic polymerization and copper-mediated radical polymerization have demonstrated excellent end-group fidelity and have functionalities that can be readily modified to a click-like reactive group (*e.g.* the termination of the anionic polymerization with ethylene oxide to yield a terminal hydroxyl,^67^ to then yield a terminal azide^77^).

Click concepts in macromolecular systems clearly must take into consideration the challenges in purification and detailed molecular characterization. For cases in which end-group fidelity is not maintained, the coupled BCPs mixture also will contain one or both of the un-coupled building blocks that can be difficult if not time-consuming to remove, unless the click-based reaction conditions and stoichiometry are defined to yield only the BCP and an easily separable building block. Furthermore, it is worth noting that not all small molecule click reactions are similarly effective in polymer systems. The radical-mediated thiol–ene reaction is an example of a reaction that has found application in small molecule and polymer modifications,^78^ but it has not been as efficient in polymer–polymer couplings. This reaction was demonstrated clearly by DuPrez and Barner-Kowollik to have very limited effectiveness for the coupling of a range of chain-end functionalized polymers.^79^ Studies such as these highlight the need for in-depth and careful characterization of such BCP reaction schemes to verify the effectiveness of the BCP formation (*i.e.* homopolymer contamination, *etc.*). Click-based approaches also have been employed in the post-polymerization modification of pre-formed BCPs. As demonstrated by Hammond and coworkers, alkyne side groups allow the creation of a versatile library of compounds from a single parent BCP through cycloadditions;^80^ however, the efficiency of the coupling reactions remains a concern when dealing with multiple reaction sites on a long-chain macromolecule.

A further challenge to be overcome, which affects all of the synthesis methods described above, is the requirement for compatible solvents for the constituent blocks of the BCP. This constraint can be especially challenging for both macroinitiation and post-polymerization coupling, and if not properly considered can lead to low yields, incomplete BCP formation, and high dispersities, reducing the sustainability of BCP generation. Two classes of macromolecules in which these issues are particularly common are organic-biopolymers, such as nucleic acid–hydrophobic polymer conjugates, and conducting-organic BCPs, given that the conducting blocks often have limited solubilities in a wide range of solvents. An additional challenge in biomacromolecule coupling is that click-based groups must not undergo reactions with non-target sites, which is a concern in polymer/protein coupling (for example, through thiol–ene chemistry, when the protein construct may contain multiple cysteine residues). Thus, although small molecule click concepts are extremely useful in coupling macromolecular systems, care must be taken to generate well-defined and well-characterized BCPs.

## Characterization of complex nanoscale assemblies

The self-assembly of complex macromolecules generated through various synthetic methods is of critical importance in the fabrication of materials targeted toward next-generation applications. Whereas conventional BCPs are described by a manageable set of parameters (typically *χN*, block volume fractions, and statistical segment length ratios), many new systems incorporate multiple blocks with various architectures, dopants, and specific interactions.^4^ These factors significantly complicate BCP self-assembly, as well as confound requisite nanoscale characterization.

### Bulk systems

For bulk systems, wide angle and small angle X-ray scattering (WAXS and SAXS), small angle neutron scattering (SANS) transmission electron microscopy (TEM) and TEM tomography, mechanical analysis, birefringence, calorimetry, and transport measurements are some of the common tools employed in materials characterization.^3^ While these techniques are extremely useful for nanostructure determination, methods such as TEM, calorimetry, and mechanical analysis are not always amenable to rapid materials discovery. Thus, more detailed and customizable characterization tools are necessary to quickly elucidate the intricate structures in complex materials for which macromolecular constituents and processing steps are chosen, not to facilitate characterization, but to enable applications. Recent advances in that direction include the emergence of resonant soft X-ray scattering (RSoXS),^81^ which can distinguish between nanoscale domains in BCPs that contain distinct chemical constituents without an over-reliance on strict electron density contrast. Another technique, energy-filtered TEM (EFTEM), has been used to probe chemical heterogeneity between domains caused by differences in block chemistries, as well as the locations of small molecule dopants.^82^ When augmented with other newly pioneered methods in soft materials, such as X-ray Photoelectron Spectroscopy (XPS) with C_60_^+^ ion sputtering, the elucidation of the spatial distributions of chemical species within multicomponent and polymer-based systems is facilitated in bulk and thin film (see below) materials.^83^ Though RSoXS, EFTEM, and XPS-C_60_^+^ sputtering provide substantial opportunities for the improved nanoscale characterization of soft materials, further progress is necessary to accurately probe three-dimensional and hierarchically-ordered nanomaterials in a rapid fashion.

### Thin films

Similar challenges and opportunities exist in the analysis of nanostructured thin film systems. Major characterization techniques include optical microscopy (OM, to analyze island/hole formation and wetting behavior), atomic force microscopy (AFM), TEM, scanning electron microscopy (SEM), grazing-incidence SAXS (GISAXS), reflectivity (neutron and X-ray), XPS, time-of-flight secondary-ion mass spectrometry (ToF SIMS), and contact angle (surface energy) measurements. While techniques such as OM enable rapid materials characterization when combined with high-throughput or gradient approaches,^84^ the remaining nanostructure investigation methods typically are time-consuming and preparation-intensive. There have been substantive recent developments in several advanced tools for expedited thin film nanoscale characterization;^85,86^ however, significant innovations are necessary to probe two-dimensional and three-dimensional structures over large areas, monitor defects, and provide real-time information during nanostructure processing. The incorporation of thermal and solvent annealing apparatuses into GISAXS and AFM experiments has begun to provide some insights into processing effects on nanostructure stability,^87^ yet the role of thermal, solvent, and surface field gradients within thin films remains an underexplored area.^24,26,88,89^ Neutron scattering techniques such as reflectometry, rotational SANS (RSANS), and multiple angle grazing incidence K-vector (MAGIK) off-specular reflectometry can provide significant insights into the thin film ordering processes,^90^ in particular, by elucidating the spatial distribution of solvents in thin films during casting and processing. Insights such as these will be extremely useful in the design of appropriate conditions to maximize the desired nanostructure ordering. Furthermore, continued development of techniques such as ultra-fast AFM and *in situ* TEM provide key opportunities for monitoring the kinetic processes that are so influential in thin film behavior.^26,85,91^

### Solution assembly

**Fig. 5:**
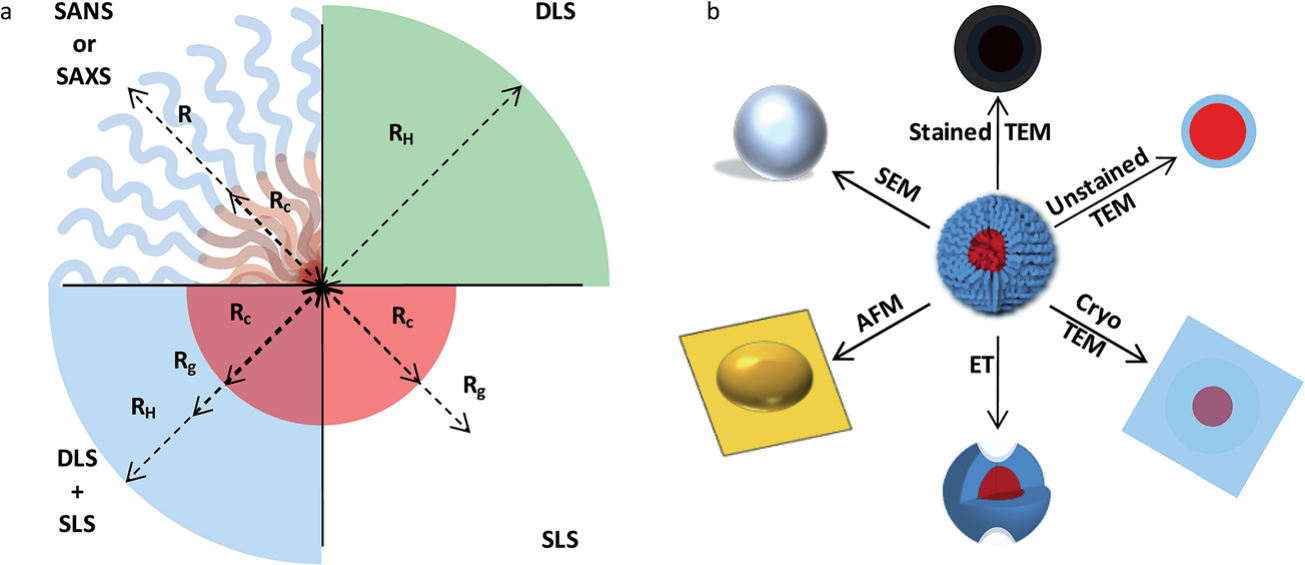
Characterization of block copolymer solution assemblies. Schematic showing (a) a subset of the structural information obtainable from various scattering techniques and (b) a subset of images extracted using different microscopy techniques for a spherical polymer micelle. Panel (a) highlights common structural dimensions that can be readily probed in a idealized spherical micelle, along with associated reciprocal-space scattering techniques; these dimensions include *R*_c_ (core radius), *R*_g_ (radius of gyration), *R*_h_ (hydrodynamic radius), *R* (micelle radius). Panel (b) illustrates complementary information that can be gleaned from real-space microscopy imaging [reprinted with permission from ref. 92 © Royal Society of Chemistry].

The characterization of solution assemblies faces similar challenges, including the impact of kinetics and processing on the overall assembly process. However, solution assemblies face the additional difficulty of a dearth of nanoscale characterization tools that can perform *in situ* analysis of macromolecular aggregates and allow for determination of key nanostructure characteristics, such as the core radius, hydrodynamic radius and/or radius of gyration of a spherical micelle (Fig. 5). While methods such as dynamic and static light scattering (DLS and SLS), calorimetry, spectroscopy (*e.g.* nuclear magnetic resonance [NMR] and Raman), and rheology are capable of following gross aggregate evolution, techniques such as SAXS, SANS, TEM, and cryogenic-TEM (cryo-TEM) are capable of probing the fine details of aggregate structures, such as core and corona density profiles.^92^ To fully understand the self-assembly process time-resolved measurements are required; however, methods such as microscopy (and scattering) are challenging to perform in this manner, largely due to the acquisition times and the larger background signals associated with probing a dilute solution environment. Additionally, approaches such as neutron scattering routinely rely on the ability to synthesize systems with the appropriate contrast (*i.e.* deuterated materials),^51^ sometimes leading to fabrication for analysis and not application. Furthermore, methods such as TEM involve sample preparations that remove the assemblies from their native environment with sometimes unintended consequences;^92^ thus, new approaches are necessary to capture the true behavior of amphipathic solution assemblies. Synchrotron radiation and pulsed neutron sources, along with stopped-flow techniques,^93^ have the potential to provide detailed nanostructure information on the time scales of interest, but again, require well-designed systems. Recent studies have shown that real-time/solution state TEM (RT-TEM) is an intriguing alternative for monitoring assembly and stability, while maintaining access to detailed structural information;^94^ however, it is worth noting that samples with sufficient contrast and suitable electron beam stability are necessary to fully take advantage of RT-TEM.

## Theory/simulation/modelling

**Fig. 6:**
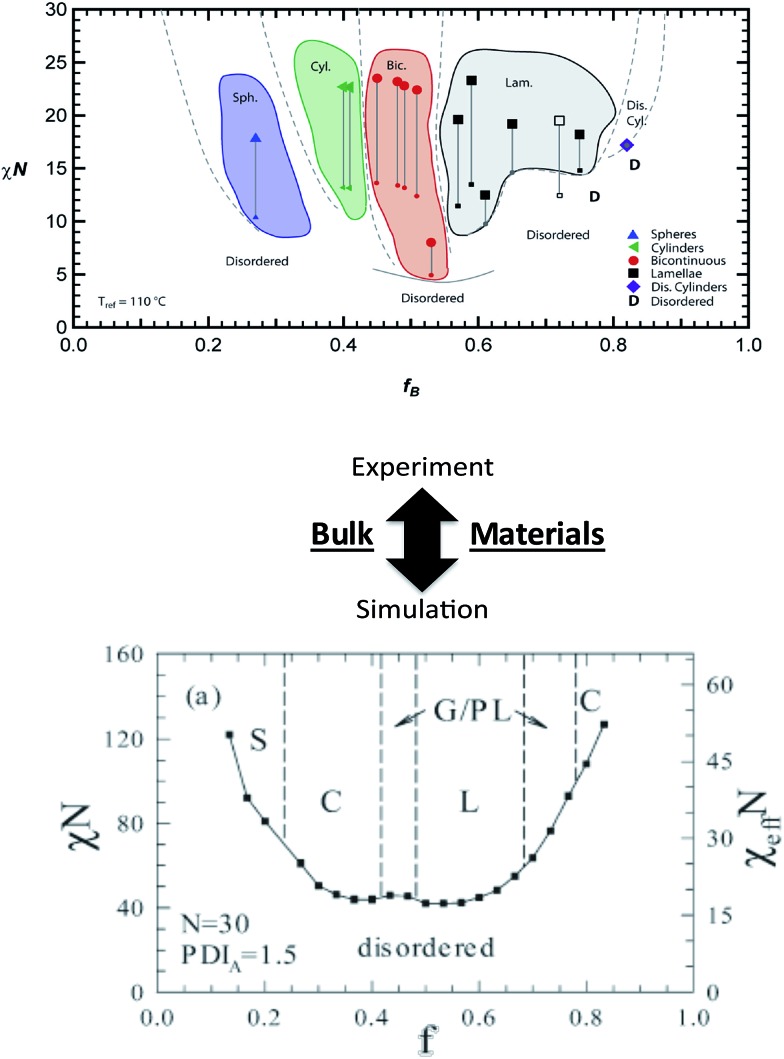
Linking theory and experiment in block copolymers. Bulk nanostructures – effects of polymer dispersity. (Top image) experimental phase diagram of a block copolymer system containing controlled dispersity [reprinted with permission from ref. 8 © American Chemical Society]. (Bottom image) Monte Carlo simulated phase diagram of block copolymer system with a similar dispersity concept [reprinted with permission from ref. 95 © American Chemical Society]. A comparison between the top (experimental) and bottom (simulated) images shows general agreement and illustrates how theory and simulations could potentially direct experimental efforts.

Numerous theoretical advances also can provide significant insights into BCP assembly and detail key information in the experimental development of new nanostructured systems. Self-consistent field theory (SCFT), density functional theory, molecular dynamics, and Monte Carlo (MC) simulations have been particularly useful in improving understanding of BCP nanostructure formation in bulk and thin films. Recent examples in bulk BCPs include the SCFT work of Hall and coworkers^65^ that examined the influence of controlled heterogeneity between homogeneous blocks (*i.e.* tapering) on self-assembly of a diblock copolymer, and the lattice-based MC simulations of Matsen and coworkers^95^ that explored the effects of controlled dispersity on copolymer phase behavior (Fig. 6). In each of these cases, the theoretical efforts complemented recent experimental work and provided tantalizing information useful in the fabrication of new nanoscale materials.^8^ Additionally, reports by Wang and coworkers^96^ (SCFT of salt-doped BCP melts) and Jayaraman and coworkers^97^ (self-consistent Polymer Reference Interaction Site Model [PRISM] theory/MC simulations of copolymer coatings on nanoparticles in homopolymer matrices) again show direct experimental links that can aid materials discovery and provide practical trajectories for experimental investigations. In addition to the powerful trends highlighted in the above studies, further theoretical advances could eventually lead to specific predictive capabilities such as exact polymer constituents, molecular weights, architectures, dispersities, grafting densities, *etc.* that would produce the desired macromolecular behavior or specified nanostructure. These capabilities would dramatically streamline synthesis and characterization and lead to informed macromolecular design.

**Fig. 7:**
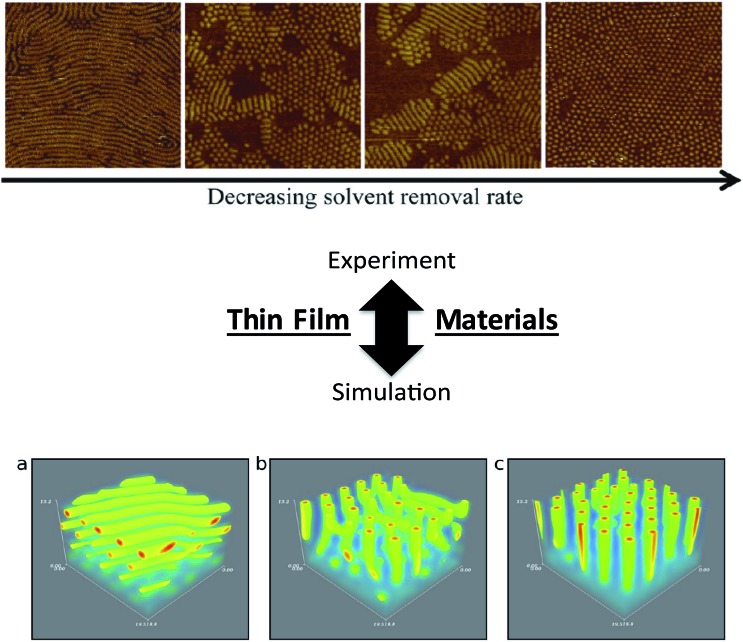
Linking theory and experiment in block copolymers. Thin film nanostructures – effects of processing on nanostructure orientation. (Top image) AFM images of parallel and perpendicular orientations of cylinders as a function of solvent evaporation rate in an ABA-triblock copolymer thin film [reprinted with permission from ref. 100 © American Chemical Society]. (Bottom image) parallel *vs.* perpendicular orientations generated from various solvent evaporation rates and solvent selectivities in BCP thin films using a dynamic extension of self-consistent field theory simulations [reprinted with permission from ref. 88 © American Chemical Society]. In both the experimental and simulation efforts, faster solvent removal (evaporation) rates led to parallel orientations of cylinders, while slower solvent removal (evaporation) rates led to perpendicular cylinders. Refinement of general experimental and simulation trends, such as the correlation between solvent evaporation and nanostructure formation, will facilitate the continued development of nanostructure/processing relationships in thin film systems.

Similar insights have been made in BCP thin films, recent examples of which include the work of Alexander-Katz and coworkers (forward SCFT simulations in an inverse design algorithm to explore topographic templates for directed self-assembly)^98^ and dePablo and coworkers^99^ (MC simulations of BCP thin films on nanopatterned surfaces) that have produced detailed information to strengthen practical understanding of directed assembly on designer substrates. Furthermore, efforts by Frederickson and coworkers examined the influence of solvent removal rate on the stability of cylindrical orientations in ultrathin films using dynamical field theory simulations,^88^ providing key knowledge that can be translated readily to experimental and application-oriented systems (Fig. 7).^100^ The above examples demonstrate significant progress in linking theoretical studies to experimental investigations, and continued efforts in understanding the effects of thin film processing and fabrication methods, substrate interactions, and influences of macromolecular architectures on kinetic *vs.* thermodynamic assembly could drive significant advances in nanopatterning and sensing platforms using BCP thin films.

Solution assembly of BCPs presents great challenges for conventional theoretical/modelling/simulation methodologies. The intrinsic need to explicitly describe key interactions from angstroms to tens of nanometers, over relevant time-scales, necessitates multi-scale approaches; however, accurate descriptions of thermodynamic and kinetic processes are hampered by the inability to simultaneously access the necessary time-scales and length-scales. Several simulation/theory efforts have made significant in-roads into the understanding of BCP behavior in solution assemblies. Notable recent efforts include the exploration of equilibrium BCP chain exchange kinetics in dilute micellar solutions,^101^ examination of the energetics of unimer insertion in concentrated micelle solutions,^102^ and combined experimental and theoretical probing of the influence of BCP molecular weight and composition on critical aggregation concentration.^103^ While these works indicate substantial progress, many challenges remain, especially related to the formation/processing of solution assemblies.^104^ One path forward is to consider approaches currently applied in protein-engineering, especially in nature-inspired materials, to reconcile the influence of thermodynamics and kinetics (and processing) on protein folding that may be particularly applicable for many solution assembled BCP systems.^105^

## Emerging directions and major challenges for block copolymer assemblies

Several challenges exist along the path toward accelerating the design of new nanostructured materials with positive societal and environmental impacts through the leveraging of continued advances in macromolecular synthesis, processing, and characterization. In particular, the desire to unlock exotic and hierarchically complex nanostructures for next-generation applications requires the multidimensional understanding of a myriad of chemistries, molecular architectures, fabrication protocols, and processing techniques.^106^ This understanding can be facilitated by additional links between experiment and theory that provide true predictive capabilities. Furthermore, although not all-encompassing, three emerging areas that have been foreshadowed by the above discussions are: *(1) the sustainable and environmentally-friendly generation and processing of materials, (2) the optimization and detailed characterization of nature-inspired materials, and (3) the influence of processing and fabrication methods on nanoscale structures, in particular, solution assemblies*.

### Sustainable and environmentally-friendly generation and processing of materials

Major efforts in the sustainable and environmentally-friendly generation and processing of materials have focused on the synthesis of nanostructured polymers from bio-based or renewable feedstocks.^107^ Many of these “green” systems have attractive functionalities (*e.g.* aldehydes, hydroxyls, and phenols) that permit the design of new monomers amenable to controlled polymerization techniques (or bio-based sources of “old” monomers) for applications such as thermoplastic elastomers, pressure-sensitive adhesives, nanocarriers for biological and catalysis applications, and blend compatibilizers; however, further efforts in cultivating sustainable and cost-effective feedstocks, achieving efficient syntheses (and purification), and obtaining suitable macroscale properties (glass transition temperature, degradation temperature, modulus, solubility, *etc.*) are necessary. As highlighted in the polymer synthesis sections, sustainable macromolecules generation also applies to the continued development of multi-component polymerization and coupling approaches that further reduce waste, solvent usage, and purification requirements.

### Optimization and detailed characterization of nature-inspired materials

As nature has become an inspiration for the exploration of self-assembled systems, bio-inspired materials embody many of the strengths and challenges in nanomaterials design. In contrast to biological polymers (such as proteins or DNA), synthetic polymers can be prepared from a much broader range of monomers, to afford polymers with a variety of structures and architectures and hence a vast range of properties and diversity of applications including diagnostics, therapeutic agent delivery, cell culture and tissue engineering, and biomaterials scaffolds and supports. However, a primary limitation of current synthetic polymers is the lack of general methods for producing precise chain structure (*i.e.* sequence control) and hence complex function (*i.e.* replication and evolution). Indeed, the development of new methods to enable the mimicking of biological function in macromolecules is an emerging area,^59^ which holds great potential for the future.

While the basics of conventional BCP synthesis were established some time ago, it is only more recently that innovations including the fabrication of polymer–peptide, polymer–protein, and other stimuli-responsive materials that take advantage of the exquisite interactions facilitated by unique molecular organization and secondary structure formation are now possible.^59^ Furthermore, forays into the mechanisms and energetics of peptide/protein folding and function have enabled the discovery of new methods to engineer macromolecules, in which the placement of individual repeat units is controlled to impart specific functions or directional interactions.^105^ This approach is key as it allows for the preparation of biohybrid BCPs with specific polymer-biomolecular attachments. However, as the precise manipulation of functional groups can have substantial effects on material efficacy in complex systems, it is crucial that researchers continue to explore the necessary structure/property relationships by fully understanding the limitations inherent in many macromolecular and nanostructure characterization techniques.

Additionally, as stimuli-responsive and targeted assemblies become *en vogue* for therapeutics, diagnostics, and imaging applications, information to correlate the placement of designer functionalities on macromolecules to their spatial arrangement in solution assemblies is increasingly vital to truly design and optimize nanoscale materials for *in vivo* applications.^44,105^ As one example, techniques such as anomalous SAXS (ASAXS) can probe this link in solution-assembled BCP systems,^108^ but ASAXS comes with the added expense of incorporating the appropriate moieties (such as selenium labelling) to ensure adequate contrast, along with the necessity of a synchrotron source; thus, theoretical complements to predict exact constituent/architecture/nanostructure/property relationships are vital.

### Influence of processing and fabrication methods on nanoscale structures

As new materials are envisioned with intricate and precise self-organization potential, the role of nanostructure fabrication and processing becomes an even greater consideration in materials development. As demonstrated in peptide and protein-based systems, thermal history, mechanical processing, and exposure to external fields and environments have substantial and irreversible effects on macromolecular assembly leading to path dependent behavior.^109^ In the case of solution assembly, information gleaned from the detailed literature on biomacromolecules on these processing effects can provide significant insights into the fabrication and stability of polymer solution constructs.^57^ Studies examining the influence formulation and processing protocols are particularly relevant in light of work describing the impact of the above variables on solution assembled nanostructure size and shape, which ultimately will control nanocarrier delivery and function. We note that fabrication and processing effects are not limited to solution assemblies and biomaterials but also are inherent in bulk and thin film assemblies as highlighted in the previous sections. Similar key challenges also are present in nanocomposites,^110^ organic electronic materials,^7^ and resins,^111^ among other arenas comprising block copolymers.

In summary, while efforts in high-throughput, automated, and gradient synthesis and characterization have accelerated materials development in conventional systems as well as the three areas highlighted above,^25,26^ the sheer diversity of possibilities necessitates the intimate interfacing of experimental designs with theory/simulation/modelling. To facilitate this meshing of theory and experiment, it is vital that theoretical/modelling efforts continue to consider relevant processing protocols and molecular architectures in designing appropriate systems. However, it is also important that experimental studies take advantage of the complete suite of synthetic, molecular characterization, and nanostructure characterization tools to fully and accurately characterize macromolecular assemblies. The complexity of the chemical and biological units and the plethora of possible building blocks of the next-generation of block copolymers stretch the limits of nanomaterials characterization, which reinforces the urgent need for enhanced theoretical-experimental methods in *de novo* materials design. Thus, by harnessing the inherent strengths of soft materials chemistry, physics, processing, and theory to generate complex nanomaterials, new systems and tools will be developed to unlock the full potential of BCPs and continue to shrink the gap between concept and application.

## Author contributions

T. H. E. and R. K. O. R. jointly discussed the content of the review and jointly wrote the manuscript.

## Conflict of interest

The authors declare no competing financial interests.
